# Selective pulsed field ablation of macroreentrant biatrial tachycardia guided by high-density mapping using a balloon-in-basket catheter

**DOI:** 10.1016/j.hrcr.2026.06.002

**Published:** 2026-06-10

**Authors:** Maciej Wójcik, Paweł Błaszkiewicz, Sebastian Wójcik

**Affiliations:** 1 Department of Cardiology, Medical University of Lublin, Lublin, Poland; 2 Wojewódzki Szpital Specjalistyczny w Białej Podlaskiej, Biała Podlaska, Poland; 3 Elkardia Lubelskie Centrum Kardiologii, Lublin, Poland

**Keywords:** Biatrial tachycardia, Pulsed field ablation, High-density mapping, Bachmann bundle, Macroreentry, Superior vena cava, Atrial tachycardia, Catheter ablation

**Key Teaching Points**

• Postsurgical atypical atrial tachycardia should raise suspicion for macroreentry, including biatrial tachycardia (BiAT), particularly when the tachycardia is sustained, has a stable cycle length, and lacks warm-up or cool-down behavior.
• High-density biatrial mapping can distinguish active biatrial macroreentry from passive activation and can identify critical interatrial limbs such as Bachmann bundle and superior vena cava (SVC)/right atrial connections.
• In this case, the circuit was most consistent with type 1 BiAT in the Kitamura classification, with left atrial/interatrial and right atrial/peritricuspid components.
• Balloon-in-basket pulsed field ablation with selective electrode-pair activation may allow broad substrate modification and focused critical-isthmus ablation in anatomically sensitive regions near the SVC, sinus node, and phrenic nerve.

## Introduction

Biatrial tachycardia (BiAT) is an uncommon form of atrial macroreentry in which both atria constitute essential components of the tachycardia circuit.^1^

Interatrial conduction may occur through preferential muscular pathways, including Bachmann bundle, and through septal or coronary sinus (CS) connections.^2^

BiAT is most often encountered in patients with previous atrial surgery or previous ablation, in whom scar and altered interatrial conduction create a substrate for macroreentry.^1^^,^^3^

Pulsed field ablation (PFA) is a nonthermal ablation modality with relative myocardial selectivity and potential sparing of adjacent structures.^4^^,^^5^

A balloon-in-basket PFA catheter with selectable electrode-pair activation permits either circumferential or focused energy delivery.^6^

We report a postsurgical BiAT in which high-density mapping identified a superior interatrial circuit involving Bachmann bundle and the superior vena cava (SVC) region and in which selective PFA terminated the tachycardia.

## Case report

A 69-year-old man with a history of coronary artery bypass grafting and aortic valve replacement in January 2025 was referred for catheter ablation because of recurrent atrial tachycardia beginning in July 2025. The arrhythmia was refractory to antiarrhythmic therapy and required repeated electrical cardioversions, with only transient restoration of sinus rhythm. He had no documented atrial arrhythmia before cardiac surgery.

**Figure 1:**
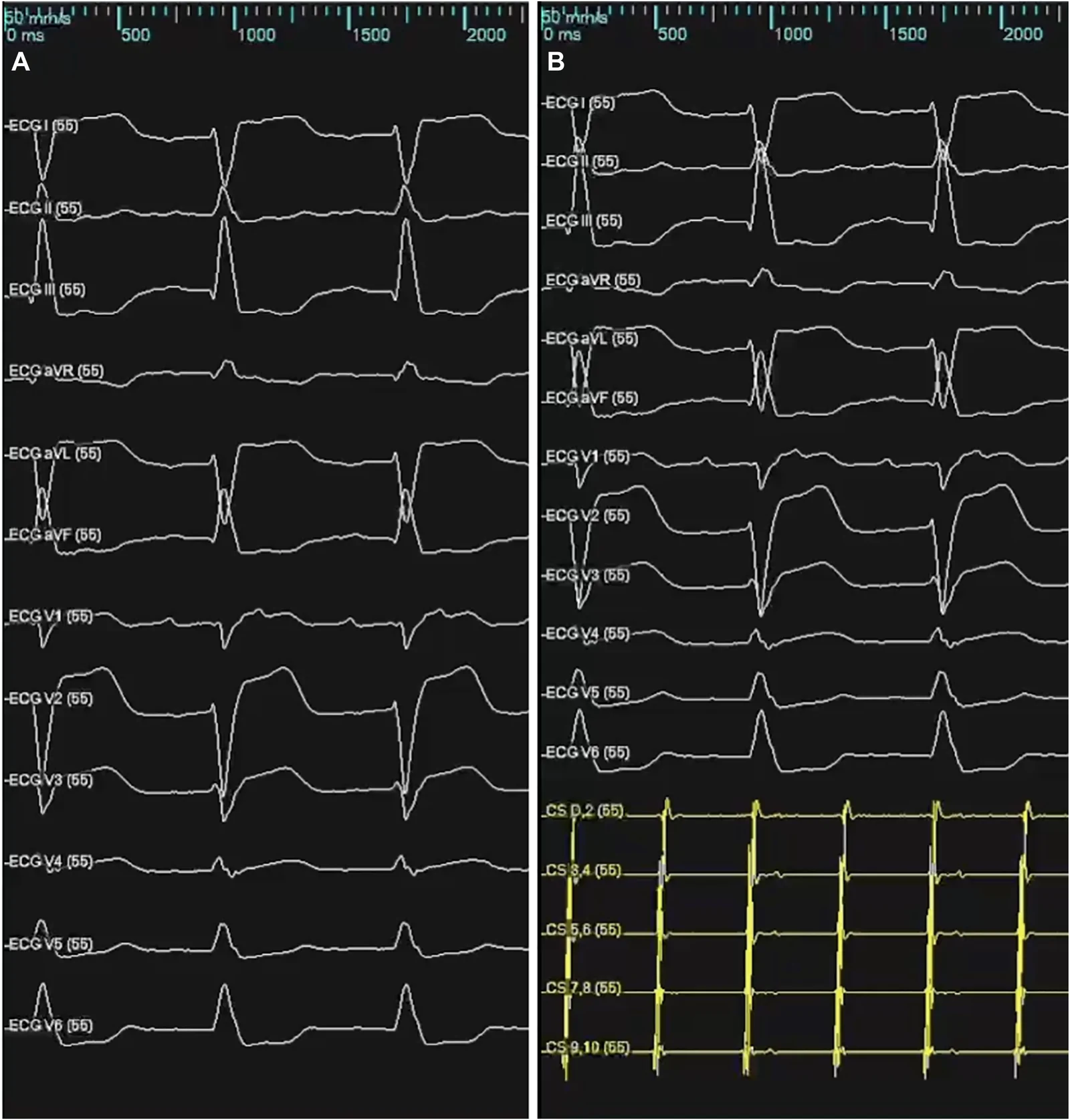
12-lead electrocardiogram and coronary sinus recording during atrial tachycardia. **A:** 12-lead electrocardiogram during atrial tachycardia (cycle length 393 ms, 2:1 atrioventricular conduction). Inferior leads (II, III, aVF): P waves are low amplitude with a late positive component and lack the sharp sawtooth pattern of typical cavotricuspid isthmus–dependent flutter; the negative or flat morphology distinguishes type 1 biatrial tachycardia from classic perimitral flutter, which often shows a positive deflection inferiorly. Lead V1: positive P wave, consistent with the left atrial component of the circuit (counterclockwise perimitral-like activation viewed from the front). Leads I and aVL: flat, low-amplitude, negative P waves, which, combined with V1 positivity, mimic perimitral flutter. Precordial leads V2–V6: low-amplitude, flat P waves, with the V1 morphology generally maintained. **B:** Simultaneous decapolar coronary sinus electrogram (*yellow trace*) showing a proximal-to-distal activation sequence, consistent with early septal/right-sided atrial activation.

The 12-lead electrocardiogram during tachycardia showed a regular narrow-QRS rhythm with 2:1 atrioventricular conduction and an atrial cycle length (CL) of 393 ms. Discrete atrial activity was visible between QRS complexes. P waves were low amplitude with a late positive component in the inferior leads, positive in lead V1, and flat to negative in leads I and aVL, without the sharp sawtooth pattern of typical cavotricuspid isthmus–dependent flutter, a combination consistent with atypical atrial tachycardia mimicking perimitral flutter (Figure 1A).

A macroreentrant mechanism was suspected clinically because of the history of recent cardiac surgery, the sustained nature of the tachycardia, stable CL, abrupt initiation and termination without warm-up or cool-down behavior, and recurrent inducibility during the electrophysiology study.

Preprocedural transesophageal echocardiography excluded intracardiac thrombus. The procedure was performed via ultrasound-guided femoral venous access. A decapolar catheter was positioned in the CS. Atrial tachycardia with CL 393 ms was reproducibly induced with atrial burst pacing at CL 350 ms and programmed atrial stimulation. CS recordings showed a proximal-to-distal activation sequence, suggesting early septal/right-sided activation (Figure 1B). The tachycardia was sustained and hemodynamically tolerated.

**Figure 2:**
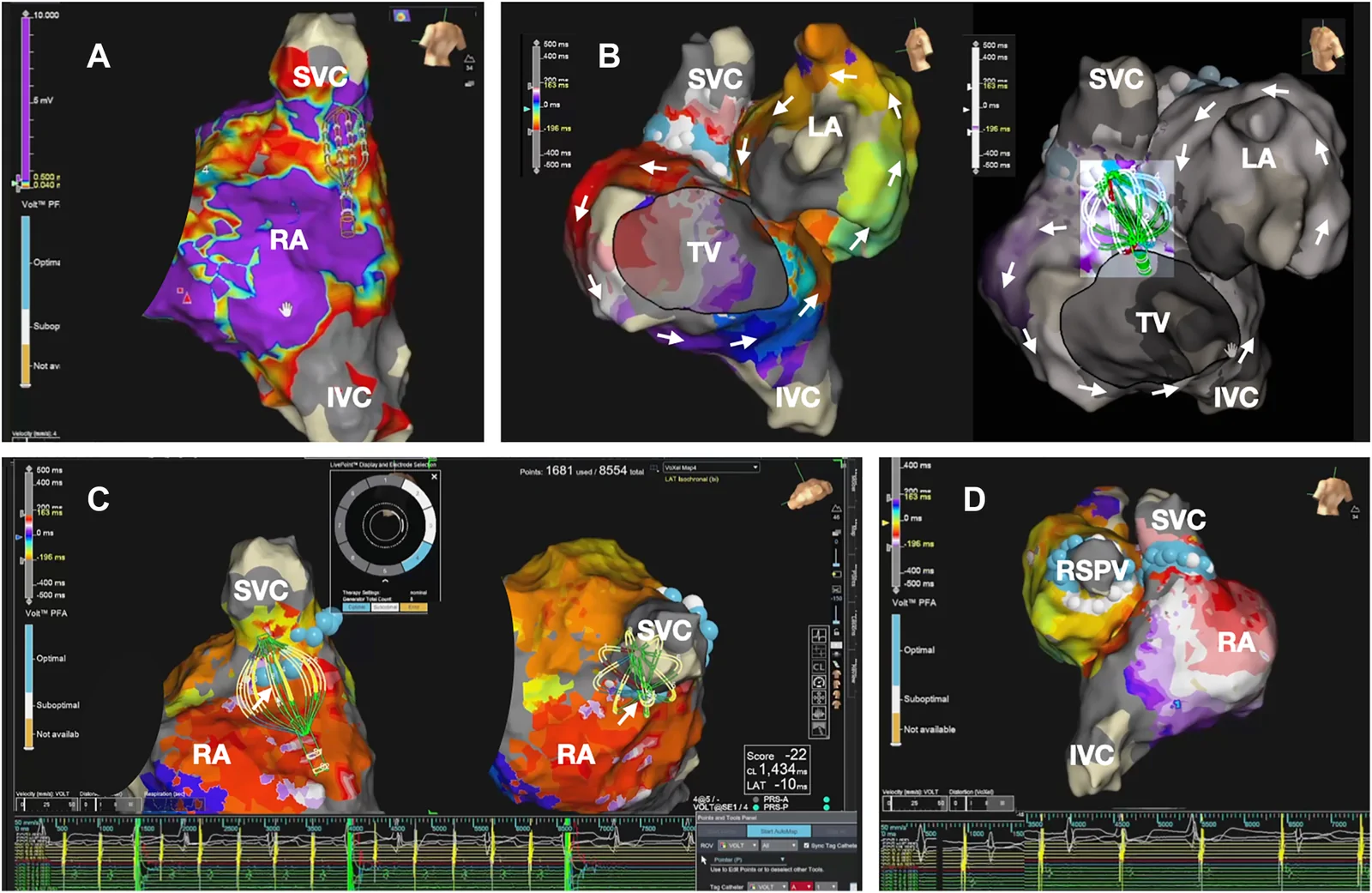
High-density mapping and selective PFA of macroreentrant biatrial tachycardia. **A:** Posterolateral RA voltage map showing a low-voltage region compatible with previous surgical access. **B:** High-density activation map showing the macroreentrant biatrial circuit. *Arrows* indicate propagation from the LA across the anterior/superior interatrial region consistent with Bachmann bundle, entry into the RA near the SVC region, continuation around the tricuspid annulus, and return toward the LA. The right panel shows the ablation catheter positioned at the functionally defined inferoseptal SVC/RA critical isthmus. **C:** Selective PFA delivered with electrodes 2–4 at the inferoseptal SVC/RA critical isthmus. Intracardiac electrograms demonstrate progressive CL prolongation from 393 to 455 ms followed by termination to sinus rhythm. *Arrows* indicate the successful selective applications leading to termination. **D:** Final lesion annotation map showing RSPV and SVC lesion sets with additional selective applications at the critical isthmus. *Blue* and *white eField tags* indicate delivered PFA applications; *AutoMark points* indicate energy-delivery sites. CL = cycle length; IVC = inferior vena cava; LA = left atrium; PFA = pulsed field ablation; RA = right atrium; RSPV = right superior pulmonary vein; SVC = superior vena cava; TV = tricuspid valve.

Biatrial high-density voltage and activation mapping were performed with a grid mapping catheter (Advisor HD Grid X mapping catheter, Abbott, Chicago, IL). Voltage mapping demonstrated preserved left atrial voltage (>0.5 mV) and a right atrial low-voltage region corresponding to previous surgical access (Figure 2A). Activation mapping accounted for the full tachycardia CL across both atria and did not identify a discrete focal earliest activation site.

Propagation mapping demonstrated a single-loop macroreentrant biatrial circuit. Activation propagated from the left atrium across the anterior/superior interatrial region consistent with Bachmann’s bundle, entered the right atrium near the SVC region, continued around the right atrium with a peritricuspid component, and returned toward the left atrium through the superior interatrial/SVC region (Figure 2B and Supplemental Movie 1). This pattern was considered most consistent with a type 1 BiAT in the schema of Kitamura et al,^1^ with participation of both the mitral/left atrial and tricuspid/right atrial components and an interatrial limb through Bachmann bundle.

The mapping catheter was then exchanged for a balloon-in-basket PFA catheter (Volt PFA system, Abbott). The ablation strategy was guided by the activation map rather than by an anatomic line alone. Because the mapped circuit traversed the superior interatrial region between the right superior pulmonary vein (RSPV) and SVC, ablation was first performed at both adjacent structures to bracket the interatrial conduction zone: 4 PFA applications were delivered for RSPV isolation and 2 applications for SVC isolation. Sinus node function and phrenic nerve capture were assessed before and after each application and remained intact.

Persistent activation at the inferoseptal SVC/right atrial junction was then targeted as the functional critical isthmus. Using selective electrode-pair activation, additional PFA applications were delivered with electrodes 2–4 positioned at this inferoseptal SVC region (Figure 2C). During energy delivery, the tachycardia CL progressively prolonged from 393 to 455 ms and then terminated to sinus rhythm. The successful site corresponded to the narrowest mapped channel between the SVC region and the right atrial septal substrate rather than to the broader anatomic SVC circumference.

After termination, sinus rhythm was stable. Programmed atrial stimulation and burst pacing failed to reinduce atrial tachycardia. A final lesion annotation map showed the delivered RSPV and SVC lesion sets and the additional selective applications at the inferoseptal SVC critical isthmus (Figure 2D). Formal entrainment mapping and formal demonstration of bidirectional block across the ablated interatrial isthmus were not performed.

The patient was discharged the next day without complications. At 164-day follow-up, he remained in sinus rhythm without recurrence of atrial arrhythmia.

## Discussion

This case emphasizes the value of mechanistic mapping in postsurgical atypical atrial tachycardia. The surface electrocardiogram did not show typical flutter morphology, and CS activation alone suggested septal/right-sided activation but could not define the circuit. The diagnosis of macroreentrant BiAT was supported by the stable tachycardia CL, reproducible induction, absence of warm-up or cool-down behavior, continuous biatrial activation spanning the entire CL, and the absence of a focal earliest activation site. The circuit was most consistent with type 1 BiAT as described by Kitamura et al,^1^ because activation involved both atria with a peritricuspid/right atrial component and a left atrial/interatrial limb using Bachmann bundle. Type 2 (right atrial septal with perimitral component) and type 3 (left atrial with right atrial septal component) BiAT were considered less likely because the right atrial limb of the circuit was peritricuspid rather than confined to the right atrial septum, and the interatrial connection was clearly superior via Bachmann bundle rather than transseptal.

The ablation target was selected from the propagation map. The decision to ablate the RSPV/SVC region rather than only the inferior septal aspect of the SVC was based on the observed superior interatrial course of activation. RSPV isolation and SVC isolation were used to bracket and modify the broader superior interatrial conduction substrate, whereas selective applications at the inferoseptal SVC/right atrial junction targeted the functional critical isthmus. Progressive CL prolongation immediately before termination suggested stepwise conduction slowing and interruption of the reentrant pathway.

Conventional radiofrequency ablation would have been a reasonable and less costly option. PFA was selected in this case because the intended lesion set involved the RSPV, SVC, and SVC–right atrial junction, regions adjacent to the phrenic nerve and close to the sinus node region. The nonthermal, relatively myocardial-selective mechanism of PFA may reduce collateral injury in such anatomically sensitive sites, although this potential advantage should not be interpreted as proven superiority for BiAT ablation from a single case.^4^^,^^5^

The balloon-in-basket design also provided a practical procedural advantage: the same catheter could deliver a broad circumferential lesion set at the RSPV and SVC and then deliver focused therapy by activating only selected electrode pairs at the critical isthmus. This combination of a large footprint with selective electrode activation may be useful when the operator must modify a broad interatrial substrate while avoiding unnecessary ablation near the sinus node and phrenic nerve.^6^

Several limitations should be acknowledged. This is a single case with mid-term follow-up. Entrainment was not performed, and formal bidirectional block across the ablated interatrial isthmus was not demonstrated. Nevertheless, the activation map accounted for the complete tachycardia CL, the site of selective PFA corresponded to the functionally defined critical isthmus, tachycardia terminated during energy delivery after CL prolongation, and arrhythmia was noninducible after ablation. These findings collectively support successful interruption of the BiAT circuit.

## Conclusion

High-density biatrial mapping can identify critical interatrial pathways in postsurgical macroreentrant BiAT. In this case, a combined PFA strategy consisting of RSPV and SVC isolation followed by selective electrode-pair ablation at the inferoseptal SVC critical isthmus terminated the tachycardia while preserving sinus node function and phrenic nerve capture. Selective PFA may be a useful option for complex atrial macroreentry involving anatomically sensitive regions, but larger experience is required before broader conclusions can be drawn.

## Disclosures

The authors have no conflicts of interest to disclose.

## Footnotes

Appendix Supplementary data associated with this article can be found in the online version at https://doi.org/10.1016/j.hrcr.2026.06.002.

## References

[1] Kitamura T., Martin R., Denis A. (2018). Characteristics of single-loop macroreentrant biatrial tachycardia diagnosed by ultrahigh-resolution mapping system. Circ Arrhythm Electrophysiol.

[2] Platonov P.G. (2014). Interatrial conduction and atrial fibrillation: what can we learn from surface ECG?. Card Electrophysiol Clin.

[3] Chugh A., Oral H., Good E. (2005). Catheter ablation of atypical atrial flutter and biatrial tachycardia. Heart Rhythm.

[4] Reddy V.Y., Neuzil P., Koruth J.S. (2019). Pulsed field ablation for pulmonary vein isolation in atrial fibrillation. J Am Coll Cardiol.

[5] Koruth J., Kuroki K., Iwasawa J. (2019). Preclinical evaluation of pulsed field ablation: electrophysiological and histological assessment of thoracic vein isolation. Circ Arrhythm Electrophysiol.

[6] Kheir J.A., Urbanek L., Urbani A. (2025). Pulsed field ablation of the cavotricuspid isthmus using a balloon-in-basket system. J Interv Card Electrophysiol.

