# Supplementary material for: Selective pulsed field ablation of macroreentrant biatrial tachycardia guided by high-density mapping using a balloon-in-basket catheter
Source: HeartRhythm Case Rep. 2026 Jun 10;12(8):871–4. doi: 10.1016/j.hrcr.2026.06.002 (PMC13492435; doi:10.1016/j.hrcr.2026.06.002)
Supplement: Supplementary file 1 [file mmc1.docx]

**Supplementary Material Legend**

Supplementary Movie 1. Dynamic high-density activation mapping of macroreentrant biatrial tachycardia.

The movie demonstrates the propagation pattern during atrial tachycardia with a cycle length of 393 ms. Activation involves a superior interatrial limb consistent with Bachmann bundle, entry into the right atrium near the SVC region, continuation through the right atrial/peritricuspid component, and involvement of the left atrial/interatrial limb. The movie complements Figure 2B by showing the dynamic propagation used to select the RSPV/SVC bracketing strategy and selective inferoseptal SVC/right atrial critical-isthmus ablation.

Abbreviations: CL = cycle length; LA = left atrium; RA = right atrium; RSPV = right superior pulmonary vein; SVC = superior vena cava; TV = tricuspid valve.
